# A multicenter, randomized controlled study on the efficacy of agomelatine in ameliorating anhedonia, reduced motivation, and circadian rhythm disruptions in patients with major depressive disorder (MDD)

**DOI:** 10.1186/s12991-023-00473-y

**Published:** 2023-11-13

**Authors:** Ping Guo, Yong Xu, Liang Lv, Min Feng, Yu Fang, Wei-Quan Huang, Shan-Fei Cheng, Min-Cai Qian, Shengliang Yang, Shi-Kai Wang, Huan-Xin Chen

**Affiliations:** 1 Department of Psychiatry, Huzhou Third Municipal Hospital Affiliated to Huzhou University, No.2088 of Tiaoxi East Road, Wuxing District, Huzhou, 313000 Zhejiang China; 2 Clinical Psychology Department, Qingdao Hospital, University of Health and Rehabilitation Sciences (Qingdao Municipal Hospital), Qingdao, 266000 China; 3 Key Laboratory, Huzhou Third Municipal Hospital Affiliated to Huzhou University, Huzhou, 313000 China; 4 Department of Anesthesiology, Huzhou Third Municipal Hospital Affiliated to Huzhou University, Huzhou, 313000 China

**Keywords:** Major depressive disorder (MDD), Anhedonia, Hypodynamia, Circadian rhythm, Agomelatine, Selective serotonin reuptake inhibitor (SSRI)

## Abstract

**Objective:**

To evaluate the clinical efficacy and safety of Agomelatine in improving symptoms in patients with major depressive disorder (MDD), providing more scientific evidence for the treatment of depression, and offering more effective therapeutic options for patients.

**Methods:**

A total of 180 MDD patients in acute phase from 10 psychiatric hospitals of Grade three in Zhejiang Province were enrolled in this 12-week study with the competitive and consecutive pattern, and they were randomized into two different groups treated with flexible-dosage antidepressants of selective serotonin reuptake inhibitors (SSRI) or agomelatine, respectively. The subjects were evaluated with psychological scales of HAMD_-17_, HAMA, SHAPS for anhedonia, MFI-20 for fatigue, PQSI for sleep quality and MEQ for disturbances in chronobiologic rhythms at baseline, 2, 4, 8 and 12-weekend points, and TESS was used for side-effect. The results were analyzed with repeated measurement analysis of variance.

**Results:**

The two groups each had 90 participants, and there were no significant differences at baseline. The scores of various assessment scales showed statistically significant time main effects during the visits (*P* < 0.01). The Agomelatine group demonstrated faster efficacy within 2 weeks, with better improvement in SHAPS, MEQ, and PSQI compared to the SSRIs group. However, the remission rate at 12 weeks was lower in the Agomelatine group than in the SSRIs group (63.3% and 72.2%), but the difference between the groups was not statistically significant. The Agomelatine group had fewer adverse reactions (14.4% and 16.7%), but there was a slightly higher incidence of liver function impairment (6.7% and 4.4%), with no statistically significant difference between the groups.

**Conclusion:**

Agomelatine, as a novel antidepressant, shows certain advantages in improving depression and anxiety symptoms and is comparable to SSRIs in terms of safety. However, its long-term efficacy and safety on MDD or other depressive subtypes still require further observation and research.

## Introduction

Major depressive disorder (MDD) is a severe psychiatric disorder characterized by high incidence, relapse rate, and disability rate [1]. More than 264 million people worldwide suffer from depression, which has become a leading cause of disability-adjusted life years [2]. Currently, even with optimal antidepressant treatments, the remission rate for MDD patients is only between 60 and 70% [3]. This suboptimal remission rate can be attributed not only to residual psychiatric [4] or somatic symptoms [5] after acute phase treatment but also to a lack of effective treatments for specific symptoms such as anhedonia and reduced motivation [6].

Anhedonia refers to the inability or diminished capacity to experience pleasure or enjoy pleasurable activities, clinically manifesting as reduced interest, joy, and emotional experience; Reduced motivation denotes decreased energy leading to heightened fatigue, diminished speech, and an overwhelming sense of exhaustion even after minimal activity. Both anhedonia and reduced motivation are core symptoms of MDD [7] and are frequently observed as residual symptoms [8]. Studies indicate that approximately 75% of MDD patients experience anhedonia [9], while 79.7% of patients suffer from diminished energy [4]. These residual symptoms not only exacerbate the severity of depression in patients [10, 11] but also negatively influence prognosis, functional recovery and treatment resistance [12, 13].

Specially, anhedonia has been reported as a linkage between the negative outcome of major depression and inflammatory markers, such as the suicidal behavior regardless of the presence or absence of psychiatric conditions. Hyperactivity hypothalamic–pituitary–adrenal (HPA) axis abnormalities, the systemic regulating factor for anhedonia, are also found to contribute the neuroinflammation and neurodegeneration and to exert an important influence on suicide [14]. Moreover, a specific focus has been proposed on a higher risk to develop antidepressant treatment resistance in subjects with enhanced neuroinflammation. Conversely, abnormally higher mean concentrations of inflammatory mediators have been found in both the periphery and brain of individuals with treatment resistance [15].

Agomelatine is a novel antidepressant with distinctive pharmacological properties and mechanisms of action, offering a departure from the conventional monoaminergic neurotransmitter theories. While it antagonizes the 5-HT_2C_ receptor, it also exerts its antidepressant effects by activating melatonin (MT) receptors MT1 and MT2 [16]. It is currently a first-line medication for the treatment of depression [17, 18]. Recent studies have found that, in addition to effectively alleviating depressive moods in patients, agomelatine shows significant efficacy in addressing specific symptoms such as anhedonia, reduced motivation, and sleep and circadian rhythm disturbances by enhancing noradrenaline and dopamine neurotransmission in the frontal cortex [19]. Chronic administration of agomelatine has been indicated to enhance neuroplasticity and neurogenesis with elevated Brain-Derived Neurotrophic Factor (BDNF) levels, particularly in the hippocampal region, which exerts a favorable efficacy in preventing relapse in patients with MDD without evidence of a discontinuation syndrome [20]. Agomelatine has also been studied for its potential effects in modulating inflammatory processes within the central nervous system, which can be implicated in the pathophysiology of depression. By agonizing melatonin receptors, agomelatine can contribute to reducing oxidative stress and protecting neural cells [21]. Given agomelatine’s action on the stress axis and circadian rhythms, it may modulate stress-induced neuroinflammation and the associated depression and cognitive dysfunction.

To compare the efficacy and safety of agomelatine and selective serotonin reuptake inhibitors (SSRIs, the standard care of MDD) in improving symptoms of anhedonia and reduced motivation in patients with MDD, various scales and measures were used to assess the remission rate and adverse reactions in patients who received different antidepressants, such as the 17-item Hamilton Depression Rating Scale (HAMD-17) for depression severity, the Hamilton Anxiety Scale (HAMA) for anxiety severity, the Snaith-Hamilton Pleasure Scale (SHAPS) for anhedonia level, the Multidimensional Fatigue Inventory-20 (MFI-20) for fatigue level, the Morningness–Eveningness Questionnaire (MEQ) for chronotype preference, the Pittsburgh Sleep Quality Index (PSQI) for sleep quality, and the Treatment Emergent Symptom Scale (TESS) for side effects. Finally, we found that agomelatine showed faster and better improvement in MDD symptoms through enhancing the neuroplasticity and adult neurogenesis, with fewer adverse reactions than SSRIs.

## Research subjects and methods

### Research subjects and procedure

**Fig. 1:**
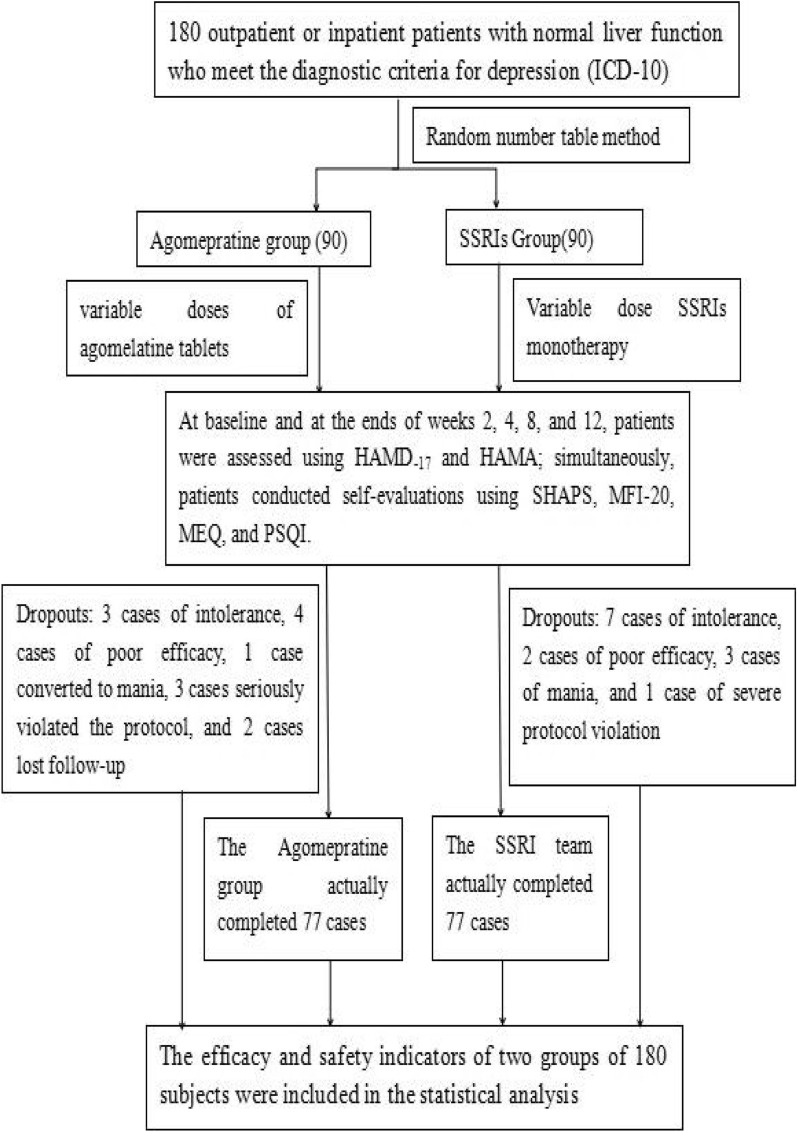
Research flow chart

Between March 1, 2019, and June 30, 2019, at ten tertiary psychiatric specialty hospitals in Zhejiang Province, based on the number of depression patient visits and referring to inclusion/exclusion criteria, a fixed quota, competitive continuous enrollment method was employed, culminating in the inclusion of 180 patients with active depression. Utilizing a computer-generated random numbering system, 180 sequences ranging from 1 to 180 were produced. Odd numbers were assigned to the agomelatine group, while even numbers were allocated to the SSRIs group. Based on the random sequence outcomes, the 180 patients were divided into the agomelatine group and the SSRIs group, with 90 patients in each group. No specific medication was prescribed for the SSRIs group, and dosages did not exceed the recommended maximum dosage outlined in the drug manuals. At baseline, demographic data such as age, gender, and Body Mass Index (BMI) were collected from enrolled patients, along with the administration of related scales for assessment. For a detailed research procedure, refer to Fig. 1.

### Inclusion and exclusion criteria, and criteria for withdrawal

Inclusion Criteria: (1) Patients diagnosed with a depressive episode based on the International Classification of Diseases 10^th^ Revision (ICD-10); (2) Diagnosis conducted using the Chinese version of the Mini-International Neuropsychiatric Interview (MINI); (3) Patients in the acute phase of a depressive episode with a total score of ≥ 17 on the HAMD-17 and a score of ≥ 3 on the Clinical Global Impression-Severity (CGI-S). Patients can be either first-episode or recurrent cases, and they should not have received antidepressant treatment or undergone Modified Electroconvulsive Therapy (MECT) or other physical therapies within the 2 weeks prior to inclusion. (4) Of Han ethnicity, both genders are acceptable, aged between 18 and 65 years; (5) Patients who have signed an informed consent form.

Exclusion Criteria: (1) Patients diagnosed with schizoaffective disorder, dysthymia, or bipolar depression; those primarily diagnosed with any other anxiety disorder within the last year; individuals with substance dependence; or those with personality disorders. (2) Carriers/patients of hepatitis B or C viruses; those with liver function abnormalities, cirrhosis, or active liver disease; (3) Patients with serious cardiac, cerebral, renal, or endocrine organ diseases or any other significant physical ailment. (4) Patients for whom previous standardized treatment with agomelatine was ineffective, or those who have been unsuccessfully treated with a full dose and full course of ≥ 2 antidepressants during the current episode. (5) Patients whose current depressive episode has lasted for more than 2 years. (6) Those with evident suicidal intentions or behaviors, with a score of ≥ 3 on the third item (suicidal thoughts) of HAMD. (7) Lactating or pregnant women, or those planning to conceive during the trial period, or those unable to employ safe and effective contraceptive measures.

Withdrawal Criteria: (1) Patient revokes their informed consent; (2) Non-compliance with study medication or therapeutic interventions during the research; (3) Persistent elevation of alanine aminotransferase (ALT) levels exceeding three times the upper limit of normal that cannot be ameliorated by hepatoprotective medications; (4) Emergence of manic shift, psychotic symptoms such as hallucinations or delusions, or disorders of consciousness; (5) Pregnancy.

This study was approved by the Ethics Committee of Huzhou City's Third People's Hospital (Ethical Approval Number: 2019 Ethics Review No. 028). All research participants or guardians are required to sign an informed consent form before participating in the study.

### Medication protocol

Patients in both groups were administered variable doses of agomelatine tablets (*Jiangsu Hansoh Pharmaceutical Co., Ltd., National Medicine Approval No. H20143375, Batch No.: 160901. Specification: 25 mg/tablet*) or SSRIs (including: Citalopram, Escitalopram, Paroxetine, Fluoxetine, Sertraline).

The study group received agomelatine tablets with an initial dose of 25 mg/day, taken once daily before bedtime. Two weeks later, based on the patient's therapeutic response and tolerance, the dosage may be adjusted to 50 mg/day, taken once daily before bedtime. The control group was treated with SSRIs. The specific choice of medication was not mandated, with all doses based on the recommended starting doses in the drug's package insert, taken once daily after breakfast. Subsequently, depending on the patient's individual response to the medication and the drug's half-life, the drug dosage can be titrated every 1–2 weeks, but the maximum daily dose should not exceed the recommended limit mentioned in the package insert.

During the study, symptomatic treatment can be administered for adverse reactions caused by the medication. Medications that were being used to treat existing somatic diseases before study inclusion can continue to be co-administered. However, during the study period, concomitant use of other antidepressants, anti-anxiety medications, antipsychotics, mood stabilizers, thyroid hormones, etc., is prohibited. The use of any systemic psychotherapy other than supportive psychotherapy is not allowed, nor is the use of MECT or other physical therapies. If necessary, short-term combined use of low-dose benzodiazepines or zopiclone is permitted but not for more than a week, to avoid affecting the observation and analysis of the patient's inherent circadian rhythm.

### Primary research tools

In addition to employing scales such as HAMD_-17_, HAMA, and PSQI, the following instruments were also utilized:

The SHAPS [22] is a tool for assessing anhedonia, encompassing domains of interest/entertainment, social interaction, sensory experience, and eating. It comprises 14 items, with a total score ranging from 14 to 56. A higher score indicates a greater degree of anhedonia.

The MFI-20 [23] consists of five dimensions: general fatigue, physical fatigue, reduced activity, reduced motivation, and mental fatigue. It encompasses 20 items, with scores ranging from 20 to 100. A higher score signifies a higher level of fatigue.

The MEQ [24, 25] is an instrument for categorizing the natural inclination of sleep/wake circadian rhythms. The questionnaire contains 19 items, with scores ranging from 16 to 86. Scores of 16–41 indicate evening-type, 42–58 intermediate-type, and 59–86 morning-type. A higher score indicates a higher degree of morningness, while a lower score indicates a higher degree of eveningness.

### Efficacy and safety assessment

At baseline and at the ends of weeks 2, 4, 8, and 12, patients were assessed using HAMD_-17_ and HAMA; simultaneously, patients conducted self-evaluations using SHAPS, MFI-20, MEQ, and PSQI. Among these, the SHAPS, MFI-20, PSQI, and MEQ scales were designated as primary efficacy indicators, while the HAMD_-17_ and HAMA scales were set as secondary indicators.

Throughout the treatment, descriptive records of various patient complaints and observed adverse reactions were maintained and evaluated using the TESS, a tool for assessing the frequency and severity of adverse reactions caused by the medication. TESS consists of 24 items that ranging from 0 to 4 scores, with a higher score indicating a more severe adverse reaction. At baseline and at the ends of weeks 4, 8, and 12, routine blood tests, urinalysis, liver and kidney functions, thyroid function, a complete set of sex hormones, and electrocardiograms were conducted.

Psychological scale assessments were carried out by two physicians from each center with intermediate or higher professional titles in psychiatry. Unaware of the patients' group allocations, they conducted cross-over blind assessments, achieving a consistency test Kappa value of 0.89.

A reduction rate of > 50% in the HAMD_-17_ total score pre- and post-treatment was deemed effective, and a HAMD-17 total score ≤ 7 was considered clinical recovery. An increase in serum ALT or AST levels beyond the upper limit of the normal range indicated liver function abnormality, while an elevation of serum ALT or Aspartate Aminotransferase (AST) greater than three times the upper limit of the normal range was considered a significant liver function abnormality.

### Statistical methods

Statistical analysis was performed using SPSS 22.0 software. The study employed a full analysis set (FAS) for data analysis, using the last observation carried forward (LOCF) method to supplement missing data, such as scores from HAMD-17, HAMA, SHAPS, etc. Quantitative data were expressed as mean ± standard deviation (*x* ± *s*). Inter-group mean differences were analyzed using the t-test. Count data and remission rates were expressed in frequencies (percentages) with inter-group rate comparisons made using the *χ*^2^ test. As baseline scores and center-specific effects could influence subsequent score changes and inter-group differences, and considering that baseline scores were assessed before the intervention, the baseline values of HAMD-17, HAMA, SHAPS, MFI-20, MEQ, PSQI scales, and individual center effects were treated as covariates. Repeated measures analysis of variance was conducted to compare group effects on the scores from these scales at various visitation points. If the sphericity assumption wasn't met, a Greenhouse–Geisser test was conducted after adjusting degrees of freedom. The test level α was set at 0.05.

## Results

### Inclusion of participants from various centers and general information and clinical assessments of depressed patients

This study involved ten qualified tertiary psychiatric specialty hospitals. A total of 180 patients with depression were planned to be included, with 90 in the Agomelatine group and 90 in the SSRIs group. During the study implementation, 26 participants dropped out after randomization at different visit points (14.45%), with 6, 11, 7, and 2 dropping out at the end of weeks 2, 4, 8, and 12, respectively. Of the dropouts, 10 were due to intolerable side effects, 6 due to inadequate therapeutic effects, 4 due to manic shifts, 4 due to prohibited medication use against the study protocol, and 2 due to relocation or school transfer. In total, 154 participants (85.55%) completed all scheduled visits. The demographic and clinical data of participants treated with different types of antidepressants are presented in Table 1.

**Table 1:** Demographic and clinical symptom assessment of depressed patients at baseline

		All Enrolled Patients		Per-Protocol Set (PPS)	
		Agomelatine Group (n = 90)	SSRIs Group (n = 90)	Agomelatine Group (n = 77)	SSRIs Group (n = 77)
*Demographic Data*					
Gender (n, %)	Males (54)	23 (42.6)	31 (57.4)	19 (42.2)	26 (57.8)
	Females (126)	67 (53.2)	59 (46.8)	58 (53.2)	51 (46.8)
First episode or relapse (n, %)	First episode (102)	49 (48)	53 (52)	43 (49.4)	44 (50.6)
	Relapse (78)	41 (52.6)	37 (47.4)	34 (50.8)	33 (49.3)
Age (years, x ± s)		42.8 ± 11.5	44.6 ± 12.6	42.5 ± 11.5	44.4 ± 14.9
BMI (Kg/m^2^, x ± s)		21.8 ± 2.8	21.5 ± 2.8	21.5 ± 3.0	21.9 ± 2.9
*Clinical Symptom Assessment*					
HAMD_-17_ (score, x ± s)		22.7 ± 6.4	22.3 ± 4.6	22.0 ± 4.3	23.1 ± 6.8
HAMA (score, x ± s)		21.0 ± 8.4	20.0 ± 8.2	21.88 ± 8.8	20.1 ± 7.8
SHAPS (score, x ± s)		45.4 ± 5.8	45.6 ± 5.7	45.8 ± 5.6	45.5 ± 5.9
MFI-20 (score, x ± s)		79.0 ± 12.1	79.2 ± 11.4	79.2 ± 12.5	79.2 ± 11.8
MEQ (score, x ± s)		51.8 ± 9.6	52.9 ± 8.8	51.6 ± 9.7	52.8 ± 9.3
PSQI (score, x ± s)		14.4 ± 3.4	14.7 ± 3.3	14.6 ± 3.3	14.3 ± 3.5

SSRIs stands for Selective Serotonin Reuptake Inhibitors; BMI indicates Body Mass Index; HAMD_-17_ is the 17-item Hamilton Depression Scale; HAMA represents the Hamilton Anxiety Scale; SHAPS is the Snaith-Hamilton Pleasure Scale; MFI-20 refers to the Multidimensional Fatigue Inventory; MEQ is the Morningness–Eveningness Questionnaire; and PSQI signifies the Pittsburgh Sleep Quality Index

The results demonstrate that among the Full Analysis Set (FAS), comprised of all enrolled patients, and the Per-Protocol Set (PPS), made up of those who met the protocol criteria, the depressive patients enrolled from various centers exhibited significant homogeneity. The demographic compositions and outcomes from clinical assessment scales presented by these two datasets were consistent. At baseline, there was no statistically significant difference in terms of age, gender, and other demographic data as well as various clinical psychological evaluation indices between the two groups of depressive patients receiving different antidepressant treatments (*P* > 0.05).

### Analysis of changes in symptoms of anhedonia and reduced motivation in depressed patients treated with different medications

As the treatment duration extended, the scores of the two groups of depressive patients treated with different medications showed a declining trend at five consecutive visit points for HAMD_-17_, HAMA, SHAPS, MFI-20, and PSQI, with the exception of MEQ which showed an increasing trend. Using the baseline scores of these scales as covariates, a repeated measures ANOVA was conducted, as shown in Table 2.

**Table 2:** Changes in Scores of Scales such as MEQ-19, HAMD_-17_ for Depressed Patients of Different Types at Various Visit Points

Scale	Group	N	Baseline	End of Week 2	End of Week 4	End of Week 8	End of Week 12	F-value and *P*-value
HAMD_-17_	Agomelatine Group	90	22.7 ± 6.4	14.3 ± 8.1	8.5 ± 6.4	6.2 ± 5.6	4.4 ± 3.9	F_T_ = 349.318, P < 0.001; F_I_ = 0.227, P = 0.923; F_T*I_ = 3.029, P = 0.084
	SSRIs Group	90	22.3 ± 4.6	13.2 ± 6.3	7.1 ± 5.2	4.8 ± 4.1	3.4 ± 3.3	
HAMA	Agomelatine Group	90	21.0 ± 8.4	14.4 ± 9.5	8.9 ± 7.4	5.5 ± 4.8	4.2 ± 4.8	F_T_ = 184.482, P < 0.001; F_I_ = 1.184, P = 0.320; F_T*I_ = 2.606, P = 0.109
	SSRIs Group	90	20.0 ± 8.2	12.2 ± 5.8	7.3 ± 5.5	5.1 ± 4.1	3.4 ± 3.3	
SHAPS	Agomelatine Group	90	45.4 ± 5.8	44.9 ± 6.3	44.9 ± 5.2	44.1 ± 3.9	40.8 ± 5.4	F_T_ = 6.897, P < 0.001; F_I_ = 0.725, P = 0.576; F_T*I_ = 0.417, P = 0.519
	SSRIs Group	90	45.6 ± 5.7	45.6 ± 4.9	44.8 ± 5.3	44.4 ± 5.8	41.5 ± 5.2	
MFI-20	Agomelatine Group	90	79.0 ± 12.1	63.7 ± 16.5	52.8 ± 15.4	46.7 ± 13.7	43.2 ± 13.9	F_T_ = 123.830, P < 0.001; F_I_ = 0.315, P = 0.868; F_T*I_ = 0.331, P = 0.566
	SSRIs Group	90	79.2 ± 11.4	62.4 ± 18.6	50.5 ± 16.4	45.8 ± 15.3	42.5 ± 15.5	
MEQ	Agomelatine Group	90	51.8 ± 9.6	53.0 ± 8.1	55.1 ± 8.8	55.7 ± 9.7	56.2 ± 10.3	F_T_ = 4.870, P = 0.001; F_I_ = 0.378, P = 0.824; F_T*I_ = 0.002, P = 0.961
	SSRIs Group	90	52.9 ± 8.8	53.1 ± 9.2	54.8 ± 9.5	55.5 ± 9.6	55.6 ± 9.9	
PSQI	Agomelatine Group	90	14.4 ± 3.4	10.3 ± 4.6	6.8 ± 3.9	6.4 ± 3.8	5.3 ± 3.3	F_T_ = 176.150, P < 0.001; F_I_ = 3.169, P = 0.016; F_T*I_ = 1.067, P = 0.303
	SSRIs Group	90	14.7 ± 3.3	10.8 ± 4.1	8.4 ± 4.3	6.9 ± 3.9	5.6 ± 3.5	

SSRIs stand for Selective Serotonin Reuptake Inhibitors; HAMD_-17_ is the 17-item Hamilton Depression Scale; HAMA is the Hamilton Anxiety Scale; SHAPS is the Snaith-Hamilton Pleasure Scale; MFI-20 denotes the Multidimensional Fatigue ; MEQ represents the Morningness-Eveningness Questionnaire; and PSQI signifies the Pittsburgh Sleep Quality Index

**Fig. 2:**
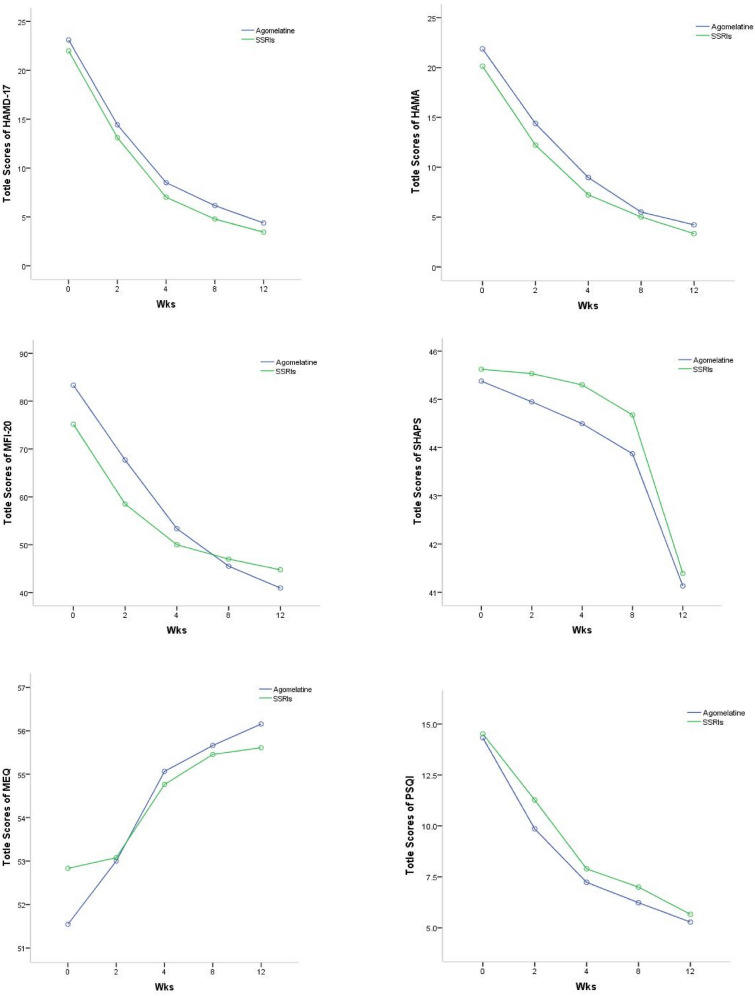
Trend chart of patients receiving different drug treatments. **A** HAMD-17 scores; **B** HAMA scores; **C** SHAPS scores; **D** MFI-20 scores; **E** MEQ scores; **F** PSQI score. HAMD-17: The 17-item Hamilton Depression Rating Scale; HAMA: Hamilton anxiety scale; SHAPS: Snaith-Hamilton Pleasure Scale; MFI-20: Multidimensional fatigue inventory-20; MEQ: Morningness–eveningness questionnaire; PSQI: Pittsburgh sleep quality index

The results indicated that the main effect of time on changes in scale scores across different treatment groups was significant (*P* < 0.01). The intergroup main effect of changes in PSQI scale scores was significant (*F*_I_ = 3.169, *P* = 0.016). Further simple effect tests revealed that the intergroup effect of PSQI scores at the end of the 4th week was statistically significant (*P* < 0.05). However, for scales other than PSQI, the intergroup main effect of score changes was not statistically significant (*P* > 0.05). The interaction of time × group for changes in all scale scores was not statistically significant (*P* > 0.05). The variations in scale scores for patients receiving different categories of antidepressant treatments across five successive visit points are illustrated in Fig. 2.

### Remission rates of both groups at the study endpoint

Using a HAMD-17 total score of ≤ 7 as the criterion for remission, the remission rates of both groups at different visit points were compared. The findings suggested that the remission rates for depressive symptoms in both groups exhibited an incremental trend across the four post-baseline visit points. Specifically, only at the end of week 2 did the Agomelatine group display a higher remission rate (20.0%) than the SSRIs group (13.3%). Nonetheless, the intergroup differences in recovery rates at each visit point were not statistically significant (*P* > 0.05), as presented in Table 3.

**Table 3:** Comparison of remission rates between the two groups after 12 weeks of treatment (n, %)

Group	N	Baseline	End of Week 2	End of Week 4	End of Week 8	End of Week 12
Agomelatine Group	90	0	18 (20.0)	31 (34.4)	45 (50.0)	57 (63.3)
SSRIs Group	90	0	12 (13.3)	32 (35.6)	52 (57.8)	65 (72.2)
			χ^2^ = 1.440 P = 0.230	χ^2^ = 0.024 P = 0.876	χ^2^ = 1.096 P = 0.295	χ^2^ = 1.628 P = 0.202

SSRIs denote Selective Serotonin Reuptake Inhibitors. After adjusting for the effects of different centers, a chi-square test was used to compare the differences in remission rates between the two groups at each visit point, all suggesting P > 0.05

### Adverse reactions

During the study, the incidence of drug-related adverse reactions in the SSRI group and the Agomelatine group was 16.7% (15/90) and 14.4% (13/90) respectively, primarily mild to moderate in nature. Both groups had 1 notable case of ALT abnormality (1.1%). Adverse reactions improved following symptomatic treatment, and there was no significant difference in the overall incidence of adverse reactions between the two groups (*χ*^2^ = 0.169, *P* = 0.681). See Table 4.

**Table 4:** Common adverse reactions during treatment in both groups

Adverse Reactions During Treatment	SSRI_S_ Group (n = 90)	Agomelatine Group (n = 90)	*χ*^2^ Value	*P*
Nausea or Vomiting	12 (13.3)	7 (7.8)	1.471	0.225
Headache or Dizziness	10 (11.1)	8 (8.9)	0.247	0.619
Anxiety or Agitation	9 (10.0)	7 (7.8)	0.274	0.600
Sleep Disturbances	8 (8.9)	5 (5.6)	0.746	0.388
Sweating	8 (8.9)	3 (3.3)	2.421	0.120
Palpitations	8 (8.9)	4 (4.4)	2.429	0.232
Sexual Dysfunction	8 (8.9)	1 (1.1)^*^	5.713	0.017
Weight Gain	7 (7.8)	1 (1.1)^*^	4.709	0.030
Diarrhea or Constipation	5 (5.6)	3 (3.3)	0.131	0.718
Liver Function Abnormalities	4 (4.4)	6 (6.7)	0.424	0.515
Tremor or Muscle Twitching	3 (3.3)	1 (1.1)	0.256	0.613
Total	15 (16.7)	13 (14.4)	0.169	0.681

SSRIs denote Selective Serotonin Reuptake Inhibitors; * represents the comparison between the Agomelatine group and the SSRIs group, P < 0.05

## Discussion

In this multicenter randomized controlled trial, we compared the efficacy and safety of agomelatine and SSRIs in improving symptoms of anhedonia, reduced motivation, and circadian rhythm disturbances in patients with MDD. We found that agomelatine showed faster and better improvement in these symptoms than SSRIs, especially in the first two weeks of treatment. However, agomelatine had a slightly lower remission rate than SSRIs at the end of 12 weeks, and a slightly higher incidence of liver function impairment. Both antidepressants were generally well tolerated and safe.

In terms of therapeutic efficacy for symptoms of anhedonia and diminished motivation, patients in this study receiving different pharmacological treatments exhibited marked decreasing trends in their SHAPS and MFI-20 scores at each subsequent visit. The main effect of score reductions over time was pronounced. However, there were no statistically significant intergroup differences at each visit, indicating that both medications effectively ameliorated symptoms of anhedonia and lack of motivation in patients with depression, consistent with some previous studies [26, 27]. Past research has suggested that Agomelatine's efficacy in treating anhedonia is superior to Venlafaxine [28] and Bupropion [29]. Similar findings were reflected in our study, with Agomelatine showing greater reductions in SHAPS scores at all visit points within 12 weeks compared to SSRIs, particularly pronounced in the initial 2 weeks. This suggests that for persistent anhedonia in patients with depression, Agomelatine can be the preferred therapeutic choice.

Regarding the onset of action in treating anhedonia and reduced motivation, the trajectory of score changes over time differed between the two groups. The pattern of MFI-20 scores aligned generally with changes in HAMD_-17_ and HAMA scores, indicating that symptoms of diminished motivation improved concurrently with the alleviation of depression and anxiety symptoms. In contrast, SHAPS scores showed a more gradual change during the initial 8 weeks and only began to rapidly improve thereafter. This suggests that the resolution of anhedonia symptoms is notably delayed compared to anxiety and depressive symptoms. Similar observations have been previously reported [30], further confirming that as a prevalent residual symptom during the acute phase of depression, the remission of anhedonia relies on continued treatment during the consolidation phase of depression. Nevertheless, compared to SSRIs, Agomelatine was able to improve anhedonia symptoms within 2 weeks, aligning with some earlier studies. Boyer et al. [30] found that Escitalopram only became effective between the 4th and 8th weeks of treatment, whereas di Giannantonio et al. [31] observed, without setting a control group, that Agomelatine improved symptoms of anhedonia in patients with depression by the second week, as identified through intra-individual pre-post comparisons.

In the realms of circadian rhythms and sleep quality, prior research has indicated that circadian rhythm disturbances are among the pivotal etiological factors for depression, with evening chronotypes facing heightened susceptibility to depressive disorders [32, 33]. Correcting these rhythms can significantly ameliorate depressive symptoms [34, 35]. This study reveals that for patients with depression undergoing varied pharmacological treatments, their PSQI scores consistently declined across successive visits. Notably, the rate of reduction was more rapid in the Agomelatine cohort, with significant inter-group score disparities emerging by the end of the 4th week. Correspondingly, MEQ scores steadily increased with each subsequent visit. While the differences between groups at each visit were not statistically significant, the elevation was more pronounced for patients in the Agomelatine group. This suggests that post-antidepressant treatment, while the sleep quality of patients with depression improves, the circadian preference of some patients’ transitions from an unhealthy evening chronotype to a healthier morning chronotype. The simultaneous modulation of circadian rhythms and enhancement of sleep quality constitutes a vital pharmacological mechanism and unique therapeutic feature of Agomelatine, as echoed by numerous prior studies [28, 36]. Additionally, the study discerned that Agomelatine’s regulation of circadian rhythms surpasses that of SSRIs and can manifest marked improvements within the initial four weeks of treatment.

In terms of drug tolerability and safety, both categories of medications exhibited commendable profiles, devoid of severe adverse reactions. Aligning with previous findings [37], the overall adverse reaction incidence in the Agomelatine cohort was lower than in the SSRI cohort (16.7% and 14.4% respectively). Significant disparities between the groups in terms of sexual dysfunction and weight implications have been documented in past reports [38, 39]. Regarding hepatic impairment, the incidence in the Agomelatine group was higher than the SSRI group (6.7% versus 4.4%), consistent with the results of a 2016 meta-analysis [40]. Hepatic impairments in both groups normalized following standard hepatoprotective interventions, without necessitating treatment discontinuation. The onset of functional impairment in the Agomelatine group was variable, with two instances emerging within each of the 1st, 2nd, and 3rd months in this study.

In terms of overall therapeutic efficacy, the Agomelatine group demonstrated a consistent decline in both HAMD_-17_ and HAMA aggregate scores across the five observational points during the study period, showcasing a pronounced time effect of score reduction. Nevertheless, inter-group differences between the two patient groups at each observational point were not statistically significant. This implies that both Agomelatine and SSRIs can effectively mitigate symptoms of depression and anxiety, yet their overarching efficacy did not exhibit discernible statistical disparities, aligning with previous findings [41–43]. However, a slight deviation from earlier studies was noted. By the conclusion of the 2nd week, the remission rate for depressive symptoms in the Agomelatine cohort (20.0%) surpassed that of the SSRI group (13.3%). Subsequent remission rates at later observation points for Agomelatine were lower than SSRIs, culminating in rates of 63.3% and 72.2%, respectively, by the 12th week. This indicates that Agomelatine demonstrates efficacy as early as within two weeks, yet its prolonged therapeutic efficacy might be slightly inferior to SSRIs. Such findings resonate with the network meta-analysis by Cipriani et al. [44], suggesting that when juxtaposed with SSRIs, Agomelatine's acute-phase efficacy for depression is subpar to Paroxetine, Fluvoxamine, Escitalopram, and Sertraline, but superior to Fluoxetine and Citalopram.

Our study has some limitations that need to be acknowledged. First, our sample size was modest and may not have enough power to detect significant differences between agomelatine and SSRIs in some outcomes. Second, we did not include a placebo group as a control group, which may introduce bias in the assessment of efficacy and safety. Third, we used a heterogeneous group of SSRIs as a comparison group, which may obscure the specific effects of each SSRI on different aspects of depression. Fourth, we followed up the patients for only 12 weeks, which may not be sufficient to evaluate the long-term effects of agomelatine and SSRIs on depression and its related symptoms. Fifth, we excluded other depressive subtypes such as dysthymia or bipolar depression from our study, which may limit the generalizability of our findings to other populations.

## Conclusion

Agomelatine is an intriguing option for patients with MDD who suffer from anhedonia, low motivation, and circadian rhythm disturbances, with generally well tolerability and safety. For clinical practice, our findings support that agomelatine can be considered as a first-line treatment for MDD or an alternative treatment for patients who are concerned about the sexual dysfunction and weight gain caused by SSRIs. Nonetheless, the long-term efficacy and safety profile of agomelatine are warranted and further evaluation for the effects of agomelatine on other depressive subtypes are needed.

## Data Availability

The datasets used and analyzed during the current study are available from the corresponding author on reasonable request.

## Abbreviations

MDD: Major depressive disorder
BMI: Body Mass Index
MT: Melatonin
SSRIs: Selective serotonin reuptake inhibitor
HAMD-17: The 17-item Hamilton Depression Rating Scale
HAMA: Hamilton anxiety scale
SHAPS: Snaith-Hamilton Pleasure Scale
MFI-20: Multidimensional fatigue inventory-20
MEQ: Morningness–eveningness questionnaire
PSQI: Pittsburgh sleep quality index
ICD-10: International Classification of Diseases 10th Revision
MINI: Mini-international neuropsychiatric interview
CGI-S: Clinical Global Impression-Severity
MECT: Modified electroconvulsive therapy
ALT: Alamine aminotransferase
AST: Aspartate Aminotransferase
TESS: Treatment Emergent Symptom Scale
LOCF: Last observation carried forward
PPS: PerProtocol Set
FAS: Full Analysis Set
